# Improvement of Performance Degradation in Synthetic Aperture Extension of Enhanced Axial Resolution Ultrasound Imaging Based on Frequency Sweep

**DOI:** 10.3390/s19102414

**Published:** 2019-05-27

**Authors:** Jing Zhu, Norio Tagawa

**Affiliations:** Graduate School of System Design, Tokyo Metropolitan University, 6-6 Asahigaoka, Hino, Tokyo 191-0065, Japan; tagawa@tmu.ac.jp

**Keywords:** axial high resolution, synthetic aperture imaging, high frame rate, carrier frequency sweep

## Abstract

We are studying a method based on the carrier frequency sweep for axial high resolution ultrasonic imaging to provide the range resolution that corresponds to the carrier wavelength. The first proposal for this type of method was based on the focused pulse transmission. Then, to improve the frame rate, the method was extended to a synthetic aperture-type method that transmits divergent pulses. While the method is effective in terms of the frame rate, degradation of the enhanced axial resolution performance is a concern. Therefore, using finite element method simulations and simple experiments, the performance of the synthetic aperture method with high axial resolution is evaluated via comparison with the original method using focused pulses. The evaluation confirmed that the performance degradation of the synthetic aperture method is caused by weakness in the transmitted wave intensity and deterioration of the phase coherence in the reception beamforming. Based on this result, we propose a method that is less affected by the latter cause and show its effectiveness.

## 1. Introduction

In high-quality ultrasound imaging, the signal-to-noise ratio (SNR) is of primary importance, and thus, a pulse compression technique (PCT) [1] that uses a frequency modulation (FM) chirp signal as the transmission pulse is commonly applied in this type of imaging. There are many studies on resolution improvement in the lateral direction as a device for receive beamforming [2,3]. However, the improvement of the resolution in the range direction is mostly due to the broadening of the frequency band, especially harmonic imaging [4,5]. In this study, we focus on the resolution in the range direction, which is usually determined by the pulse width that is to be transmitted. By using a pulse composed of one cycle of a sine wave, the pulse width is narrowed, and the axial resolution is improved, but the SNR reduced. Therefore, a pulse composed of a plurality of sine waves is often used. From this standpoint, we are studying a method to realize the resolution corresponding to the wavelength of the carrier wave.

In order to distinguish the reflections from different scatterers in the echo, use of the phase that is calculated from the in-phase (I) component and the quadrature (Q) component, which form the baseband representation of the echo, can be considered. However, the phase value for a single scatterer reflection contains a spread from the transmission pulse width, and it is difficult to use this phase to distinguish reflected waves that occur within the transmission pulse width. To resolve the ambiguity of reflected wave separation using a single phase, we focused on observation of the phase change with respect to multiple frequencies. Reflected waves at different times (i.e., at different distances) undergo different phase rotations for the same frequency change, and this can be used to separate the reflected waves. Using this strategy, we initially proposed an enhanced axial resolution method called the super-resolution FM-chirp correlation method (SCM) [6], which transmits and receives multiple times per line while presuming that a focused beam is transmitted. Here, we use the term super-resolution to refer to resolution that is better than the envelope width of the pulse; more specifically, the term refers to resolution that is comparable to the extent of the carrier wavelength.

If *K* is the number of transmissions of varying frequencies and *N* is the number of lines that constitute the image, the SCM must perform *N* × *K* transmission and reception processes. This leads to an increase in throughput and greatly restricts the frame rate. In addition, research into synthetic aperture imaging (SAI), in which divergent waves are transmitted, is being pursued actively to improve the frame rates for general B-mode imaging applications [7,8]. Because SAI can perform imaging with high frame rates, it is useful for accurate heart disease diagnosis. Recently, several studies have used a noted compressed sensing strategy to improve the frame rate of SAI [9]. We attempted to solve the multiple transmission problem of the SCM described above by applying SAI to the SCM. As a result, a synthetic aperture version of the SCM, called SA-SCM, was introduced [10], but the resulting degradation of the enhanced axial resolution performance has not been reported in detail. It is well known that the processing costs required for SCM imaging can be reduced considerably using the SA technique.

However, because delay-and-sum (DAS) beamforming using divergent waves makes the carrier waves remarkably incoherent and also weakens the echo intensity, the deterioration that occurs in the image quality of the SA-SCM becomes a concern. In this paper, we evaluate the degree of the performance degradation that occurs when using the SA-SCM, compare it with that of the SCM quantitatively, and reveal the cause of this degradation via a finite element method (FEM) simulation. In this evaluation process, we propose a method to suppress this deterioration. Furthermore, through simple experiments, we confirm that the proposed method is effective in practical applications.

This paper is organized as follows. We begin by describing both the SCM and the SA-SCM in the next section; we then present the methods and conditions used for the simulations and the experiments in Section 3 and give the simulation results and the experimental results in Section 4. Finally, the results are discussed and the conclusions are presented in Section 5.

## 2. Axial High Resolution Method Based on Frequency Sweep

### 2.1. Concept of Axial Resolution Enhancement Using Carrier Wave Phase Information

We now describe the principle of the SCM and the required procedure in detail. We transmitted an FM-chirp pulse s(t)=Re[x(t)ejω0t] with a center frequency of $\omega_{0}$ and received the echo signal y(t) in the radio-frequency (RF)-band from *D* point scatterers, which can be expressed mathematically as:

(1) $$y\left(t\right)=\int_{-\infty }^{\infty }h\left(\tau \right)s\left(t-\tau \right)d\tau ,$$

(2) $$h\left(t\right)=\sum_{i=1}^{D}h_{i}\delta \left(t-\tau_{i}\right),$$

where $\left\{h_{i}\right\}$ is the set of all scatterer reflectances, $\left\{\tau_{i}\right\}$ the set of the propagation delay times for the echoes of all scatterers, and δ(·) the Dirac delta function. In this formulation, the frequency characteristics of both the transducers and the propagation paths are omitted. The received FM-chirped echo is expressed in the form of a baseband in-phase/quadrature (IQ) signal ν(t), and a compressed signal is formulated as z(t), as follows:

(3) $$\nu \left(t\right)=\sum_{i=1}^{D}h_{i}x\left(t-\tau_{i}\right)e^{-j\omega_{0}\tau_{i}}+n\left(t\right),$$

(4) $$z\left(t\right)=\sum_{i=1}^{D}h_{i}r\left(t-\tau_{i}\right)e^{-j\omega_{0}\tau_{i}}+m\left(t\right),$$

where r(t) is the auto-correlation function of x(t) in the baseband, which has real values, and z(t) is the complex-valued delay profile. This model of the received signal is within the framework of the finite rate of innovation (FRI) [11]. This echo formulation is suitable for spatially-discrete scatterers. It is more appropriate to model small scatterers in living tissue to be distributed at all locations, and we also examined methods based on such a formulation [12].

**Figure 1:**
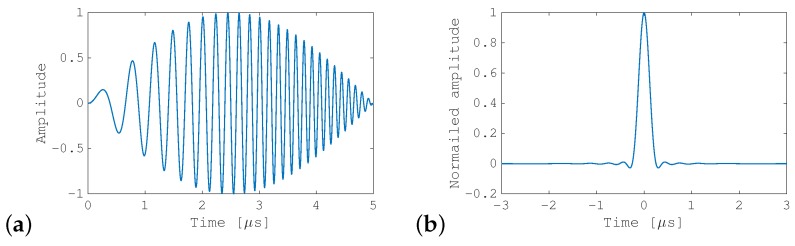
Example of frequency modulation (FM)-chirp pulse compression: (**a**) transmitted signal *s*(*t*) with a bandwidth of 8 MHz and with Hanning window apodization; and (**b**) absolute value of the compressed signal *r*(*t*).

The observation noise n(t) was assumed to be Gaussian white noise with variance $\sigma^{2}$, and m(t) is thus the complex-valued cross-correlation of x(t) with n(t). An example of FM-chirp pulse compression is shown in Figure 1.

Equation (4) shows that the echo from each scatterer has a phase that is dependent on both the carrier frequency *ω*_0_ and the scatterer’s position. Therefore, by receiving the echoes of the different carrier frequencies, it becomes possible to obtain the scatterer separation based on the phase information. This also means that the resolution that corresponds to the wavelength of the carrier wave becomes possible.

We considered a complex function space that is composed of ensemble sets of {z(t)} that are obtained by varying $\omega_{0}$. The function $\sum h_{i}r\left(t-\tau_{i}\right)e^{-j\omega_{0}\tau_{i}}$ multiplied by an arbitrary complex number exists in the same one-dimensional subspace within the complex signal space. Therefore, when *ω*_0_ is changed, such conditions may be satisfied by accident, which is a state of coherence. If this is not avoided, the echoes from the different scatterers cannot be separated in the signal space. For that purpose, it is effective to randomly change *ω*_0_ in small increments. On the other hand, for distinguishing nearby scatterers, a wide range sweep of *ω*_0_ is required. Note that this coherence problem cannot be avoided by multiple transmissions with different phases instead of different frequencies.

### 2.2. Super-Resolution FM-Chirp Correlation Method

Various methods that use this phase information have been considered. In this study, we simply applied the multiple signal classification (MUSIC) algorithm [13]. To provide a discrete representation, we defined the compressed echo vector $z\equiv {\left[z\left(t_{1}\right),z\left(t_{2}\right),\cdots ,z\left(t_{M}\right)\right]}^{⊤}$, the steering vector $r_{i}\equiv {\left[r\left(t_{1}-\tau_{i}\right),r\left(t_{2}-\tau_{i}\right),\cdots ,r\left(t_{M}-\tau_{i}\right)\right]}^{⊤}$ to indicate the compressed echo of the ith scatterer, and the noise vector $m\equiv {\left[m\left(t_{1}\right),m\left(t_{2}\right),\cdots ,m\left(t_{M}\right)\right]}^{⊤}$, where *M* is the number of times sampling is performed. Here and throughout the paper, the superscript ⊤ indicates the transpose of a vector or matrix. Using the array manifold matrix $\Gamma \equiv [r_{1},r_{2},\cdots ,r_{D}]$ and the gain vector $g\equiv {\left[h_{1}e^{-j\omega_{0}\tau_{1}},h_{2}e^{-j\omega_{0}\tau_{2}},\cdots ,h_{D}e^{-j\omega_{0}\tau_{D}}\right]}^{⊤}$, ***z*** and its variance-covariance matrix ***R*** can be formulated as follows:

(5) z=Γg+m,

(6) $$R=\Gamma G\Gamma^{⊤}+R_{n},$$

(7) $$G\equiv E_{\omega_{0}}\left[gg^{H}\right],$$

(8) $$R_{n}\equiv E_{n}\left[mm^{H}\right]=\sigma^{2}R_{0},$$

where $E_{\omega_{0}}\left[\cdot \right]$ and $E_{n}\left[\cdot \right]$ are the expectation operators with respect to *ω*_0_ and with respect to n(t), respectively, when it is assumed that the echoes and the observation noise are statistically independent. The symmetrical matrix $R_{0}$ consists of r(t), and its (k,l)th element is $r(t_{k}-t_{l})$. The superscript *H* represents the complex conjugate transpose.

The SCM is reliant on the generalized eigenvalue problem that can be defined using the eigenvalues $\left\{\lambda_{i}\right\}$ and the eigenvectors $\left\{e_{i}\right\}$, which obey:

(9) $$Re_{i}=\lambda_{i}R_{0}e_{i},\;i=1,2,\cdots ,M.$$

When M>D, the column vectors of **Γ** are linearly independent, and thus, the rank of $R-R_{n}=\Gamma G\Gamma^{H}$ is *D*. Therefore, ***R*** has *D* generalized eigenvalues that are greater than *σ*^2^ and *M* − *D* generalized eigenvalues that are equal to *σ*^2^. The set of *D* eigenvectors ${\left\{e_{i}\right\}}_{i=1}^{D}$ that corresponds to the *D* largest eigenvalues spans the signal subspace. The remaining *M* − *D* eigenvectors ${\left\{e_{i}\right\}}_{i=D+1}^{M}$ thus span the noise subspace, which does not contain signals. The noise subspace is located orthogonal to the steering vector that corresponds to the true delay time of the echo. To estimate the true delay when using the MUSIC algorithm, the orthogonality between the steering vector and the noise subspace can be evaluated by varying the delay time of the steering vector as per a super-resolution delay profile $S(t_{i})$, which is defined as:

(10) $$S\left(t_{i}\right)\equiv \frac{r_{i}^{H}R_{0}^{-1}r_{i}}{\sum_{j=D+1}^{M}{\left|r_{i}^{H}e_{j}\right|}^{2}},$$

If $t_{i}$ matches the actual scatterer position, the corresponding $r_{i}$ is then orthogonal to ${\left\{e_{j}\right\}}_{j=D+1}^{M}$, and the denominator of Equation (10) thus becomes small.

In this scheme, *D* must be the number of scatterers, and in practical applications, e.g., Akaike’s information criterion (AIC) [14,15,16] or the minimum description length (MDL) criterion [17,18] are used to determine *D*. In estimating ***R***, estimation of ***R**_n_* can be stabilized by performing multiple transmissions, known as snapshots, which all have the same frequency. However, when the real-time processing requirements are considered, it is better that the number of transmission/reception operations be small; therefore, in this study, we omitted the snapshot step. In addition, to prevent artifacts being caused through periodicity, we changed the transmission wave frequency at random. Using the *K* transmissions with the randomly-shifted frequency band, we then estimated ***R*** as an ensemble average of $\hat{R}=\left(\sum_{k=1}^{K}z_{k}z_{k}^{H}\right)/K$. Here, $z_{k}$ represents a compressed echo vector that corresponds to the kth transmission. As described in Section 1, when observing multiple IQ signals *z* while varying the frequency, the amount of available information increases. This information is used effectively in the estimation of ***R*** via the MUSIC method. The waves that are reflected by each scatterer cause different phase rotations that are dependent on the frequency change, thus meaning that they are linearly independent as a complex signal; and *D*, which is the signal subspace dimension, is equal to the number of scatterers. However, if multiple transmissions are performed with different phases rather than different frequencies, the same phase rotation occurs simultaneously for all echoes from each scatterer, and because these echoes only differ by complex number multiples, they can be regarded as one reflected wave. This is a coherence state where the dimension of the signal subspace is one, i.e., *D* = 1.

### 2.3. Extension to Synthetic Aperture Imaging

**Figure 2:**
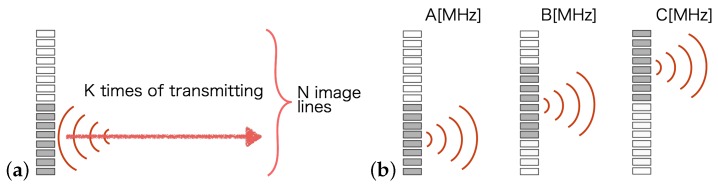
Transmission procedures for super-resolution FM-chirp correlation method (SCM) and synthetic aperture SCM (SA-SCM): (**a**) focused pulse transmission for SCM; (**b**) unfocused pulse transmission for SA-SCM.

The SCM performs enhanced axial resolution processing of each imaging line. Therefore, it is necessary to transmit multiple FM chirp pulses with different frequency bands in each direction that correspond to an imaging line; see Figure 2a. If an image consisting of *N* lines and *K* transmissions is formed for each direction, then *N* × *K* transmissions must be made to generate the complete image. This greatly reduces the frame rate of the moving image obtained. To reduce the number of transmission and that of reception processes that are required, the SCM is extended to an SAI-based version, called SA-SCM. In SAI, unfocused pulses are transmitted over a wide range from the sub-aperture elements (Figure 2b), and for each of these transmission events, echoes from the entire imaging area are received simultaneously by all elements. Dynamic focusing is performed, e.g., using DAS beam forming. By randomly changing the frequency band for each transmitted FM-chirp pulse in the SAI process, the total number of transmissions can be reduced. The *N* × *K* times transmission events in the SCM described above are reduced to *K* times transmission events in the SA-SCM. The echo signals with different frequencies that were obtained by dynamic focusing processes corresponding to each line were used as the inputs to the SCM.

To avoid frequency deviations related to the positioning of the sub-aperture used for transmission, a frequency band was assigned randomly to the position of this sub-aperture. For enhanced axial resolution imaging, the SCM algorithm uses the carrier’s phase information along the imaging line. The carrier that is contained in the compressed echo signal may be disturbed by dynamic focusing and this would cause enhanced axial resolution performance degradation.

### 2.4. Implementation Summary

This subsection summarizes the implementation of our axial high resolution method described above.

1. Pre-processing
  (a) Calculation of transmission signals
    A plurality of FM chirp signals used for transmission was calculated while randomly changing the center frequency. It is desirable that the variation width of the center frequency be wide and the variation step be small. Both are determined in consideration of the characteristics of the ultrasonic probe.
  (b) Calculation of steering vectors
    A steering vectors {***r**_i_*} corresponding to the reference FM chirp transmission signal was calculated based on the IQ representation of the autocorrelation function of the FM chirp signal. Here, *i* refers to the time shift corresponding to all sampling times.
2. Axial high resolution imaging process in each frame
  (a) Measurement
    While the transmit sub-aperture is shifted over the array, the divergent wave is transmitted and the echoes are received by all elements. Each transmission uses an FM chirp signal with a different center frequency calculated above.
  (b) Post-processing
    - Dynamic focusing
      DAS beamforming is applied to the echoes received at all elements for each transmission to calculate RF signals corresponding to each line of the image. As a result, as many RF signals as the number of transmission signals are obtained for each line.
    - Pulse compression
      Each RF signal is subjected to pulse compression processing using the corresponding transmitted FM chirp signal as a template.
    - Conversion of the RF echo signal to the IQ echo signal
      Each compressed echo signal was converted to an IQ signal *z* by quadrature detection using its carrier frequency, which is the center frequency of the original FM chirp signal.
    - Calculation of $\hat{R}$ and its eigenvalues and eigenvectors
      For each image line, calculate $\hat{R}$ by the ensemble average of $\left\{z_{k}\right\}$ (*k* indicates the kth transmission, i.e., the kth carrier frequency). Then, the eigenvalues and eigenvectors were calculated.
    - Calculation of axial high resolution signal
      For each image line, using *D* determined in advance, the super-resolution delay profile $S(t_{i})$ in Equation (10) was computed at each sampling time $t_{i}$.

## 3. Methods for Simulations and Experiments

### 3.1. Performance Analysis Using Simulations

**Figure 3:**
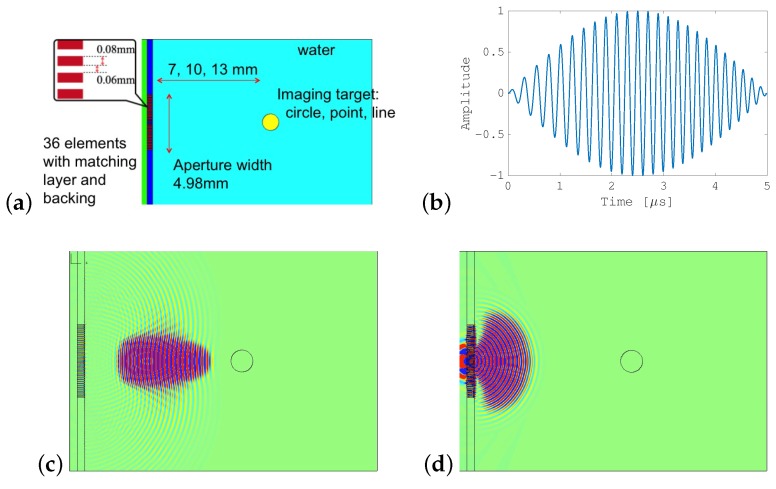
Simulation conditions: (**a**) simulation model; (**b**) transmitted FM chirp pulse with the Hanning window; (**c**) propagation of the focused beam used for SCM; (**d**) propagation of the unfocused beam used for SA-SCM.

We examined the performance of the SA-SCM via two-dimensional simulations using PZFlex (Weidlinger Associates), which is a standard FEM program for ultrasound propagation. Specifically, a performance degradation comparison with the SCM was carried out in detail. Figure 3a shows the simulation model, in which water with speed of sound of 1500 m/s and with attenuation of 0.002 dB/cm/MHz were used as a propagation medium, and an object with density of 900 kg/m^3^ and with speed of sound of 2000 m/s was the target to be imaged. A linear array transducer containing 36 elements with an element width and spacer width of 0.08 mm and 0.06 mm, respectively, is located on the left side of the figure. This transducer was provided with an appropriate matching layer and suitable backing material. Under the simulation conditions, the FEM mesh was generated such that the number of spatial samples per wavelength was at least 15, while the Courant number was set at 0.95; the time rate was set to approximately 400 MHz based on these parameters. The absorbing boundary perfectly matched layer (PML) was used as the boundary condition of the whole calculation area.

To transmit divergent pulses required for the SA-SCM, a sub-aperture composed of eight elements was used, while its position was shifted in the whole array. The transmitting focal point was −0.28 mm mm with respect to the sub-aperture width of 1.12 mm. Additionally, for the SCM, the pulse that was focused on the target object and was transmitted by all elements in the entire array. Figure 3c,d show simulations of the focused and unfocused beam transmission processes. We compared the performances of the SCM and the SA-SCM with respect to the following four items:

1. General performance
2. Effect of target position
3. Effect of transmission path
4. Effect of divergent wave

The parameter settings for the FM-chirp transmitted pulse are listed in Table 1. To suppress distortion of the transmission, it was necessary to ensure that the frequency band of the transmission pulse remained within the effective frequency band of a transducer. Therefore, the FM-chirp pulse’s frequency band was set to a relatively narrow 2 MHz in the simulations. The center frequency was changed randomly from 4–6 MHz using pseudo-random numbers with a uniform distribution. The center frequency of the FM chirp signal refers to the center frequency of the frequency sweep. Thus, for example, if the center frequency is chosen to be 4 MHz, the frequency of the up-chirp signal is swept between 3 MHz and 5 MHz. The amplitude value was set at 1 V throughout these simulations. While the signal subspace dimension *D* is an important parameter and requires careful setting for actual applications, five was used as a default value for *D* in this simulation.

### 3.2. Practical Evaluation via Experiments

In the experiments, the transmission and reception sequences were generated using an experimental platform for medical ultrasound (RSYS0003, Microsonic Inc., Tokyo, Japan) with a sampling rate of 31.25 MHz. The number of transducer elements used for both transmission and reception was 64, while the element pitch was 0.315 mm. A linear array probe (T0-1599, Nihon Dempa Kogyo Co., Ltd., Tokyo, Japan) was also used. This probe’s center frequency was 7.5 MHz, and its specific bandwidth was 70%. The signal processing required was performed offline by using MATLAB software with an Intel^®^ core i7-4770 central processing unit (CPU).

The amplitude value was set at 15 V, which was higher than that used for the simulations in Section 3.1 to avoid the effects of echo attenuation. Divergent waves were transmitted using a sub-aperture composed of eight elements with a focal point of −0.63 mm mm with respect to the sub-aperture width of 2.52 mm. Because the probe element spacing was wider than that used in the simulations, it is likely that grating lobes would be formed. Considering the range in which grating lobes were considered at an angle ±*θ* with respect to the front direction of the array transducer, the distance between adjacent transducers was *d*, and the wavelength in the propagation medium (usually water) was *λ*. The condition that did not occur is given by the following equation.

(11) $$d<\frac{\lambda }{1+\sin\theta }.$$

Therefore, for the frequency of 5 MHz in the range of ±45°, grating lobes were not generated for both transmission and reception if *d* < 0.18 mm. In the probe used in this experiment, *d* = 0.315 mm, so if the frequency is about 3 MHz or less, grating lobes will not occur. The frequency band of the FM chirp pulse that was used in the experiment was still set at a relatively narrow 2 MHz, as described in the simulations. While the frequency band that was used was not the most effective band for the probe, it was confirmed that both transmission and reception could be performed as required.

**Figure 4:**
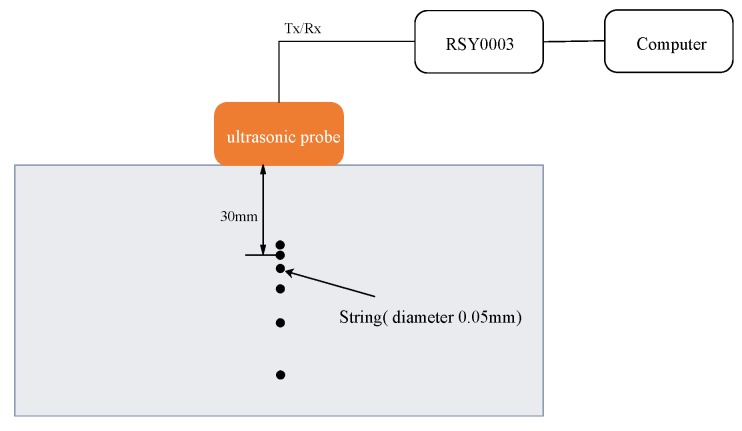
Experimental setting for the soft tissue-mimicking phantom.

We present the experimental results obtained using a soft tissue-mimicking phantom (US-2 multi-purpose phantom N-365; Kyoto Kagaku Co., Ltd., Kyoto, Japan), with a speed of sound of 1432 m/s and attenuation of 0.59 dB/cm/MHz. As shown in Figure 4, the phantom included six string wires, each of which had a diameter of 0.05 mm. The distances between these wires were 0.5, 1.0, 2.0, 3.0, and 4.0 mm, as measured from the side that was closest to the phantom’s surface. The transmission waveform, including the frequency band, was also the same as that in the simulation, but because a large number of targets was used in this experiment, it was necessary to increase the signal subspace dimensions in the SCM processing, meaning that it was thus also desirable to increase the number of transmission and reception processes. Therefore, in the phantom experiment, the number of transmission processes was increased to 25.

## 4. Results of Simulations and Experiments

### 4.1. Results of Simulations

#### 4.1.1. General Performance

**Figure 5:**
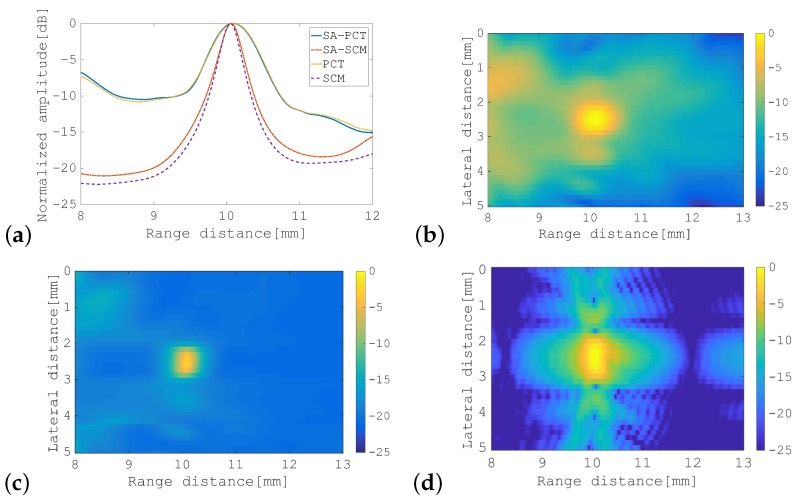
Results for the circular target with a diameter of 0.2 mm: (**a**) cross-sectional profiles of the SA-pulse compression technique (SA-PCT), the SA-SCM, focused beam PCT, and the SCM; (**b**) B-mode image of the SA-PCT; (**c**) B-mode image of the SA-SCM; (**d**) B-mode image by SA using one cycle plane wave. The color bar shows the dynamic range in dB.

First, we confirmed the performance of the SCM strategy using a circular target. Figure 5a shows the results in the form of a cross-sectional profile along the horizontal center line that passes through the object (Figure 3a), and an object with a diameter of 0.2 mm (i.e., less than the wavelength) was located at a distance of 10 mm away from the transducer. In this figure, the four results are plotted together and are numbered as follows: (i) represents SA-PCT, in which pulse compression was applied to the echoes that were received by each transducer and the SAI process was then performed on these compressed echoes for all transmissions; (ii) represents the SA-SCM; (iii) represents the normal PCT; and (iv) represents the SCM. For the SCM and PCT, the calculations were performed along the horizontal center line only, and the transmitted FM chirp pulse was focused on the object position. For the SA-PCT and the SA-SCM, all the lines, constituting the entire image, were calculated, and the results are as shown in Figure 5b,c, respectively. These two images demonstrate that the resolution in the range direction was greatly improved by the SCM processing. In addition, the performance of the SA-SCM appeared to be slightly lower than that of the SCM. In the following subsections, we investigated the causes of the performance degradation in the SA-SCM.

We used FM chirp signals to avoid SNR degradation due to divergent wave transmission. On the other hand, a method of improving the frame rate while maintaining the SNR by transmitting a one-cycle plane wave using all elements has been proposed [19]. Therefore, a plane wave consisting of one cycle of 5 MHz was transmitted 11 times, changing the direction from −25° to +25° in 5° increments with respect to the front of the probe, to generate a B-mode image. The results are shown in Figure 5d. Since the SCM is a non-linear process, it cannot be compared in the dynamic range as it is, but the −3 dB pulse width of the SA-SCM was about 0.25 mm, whereas it was about 0.35 mm in the result of plane waves. This also confirms the performance of the resolution of this method.

#### 4.1.2. Effect of Target Position

In this subsection, we compare the performances of the methods with respect to the different target positions when using a point scatterer (Figure 3a) that consists of four meshes in the FEM calculations.

**Figure 6:**
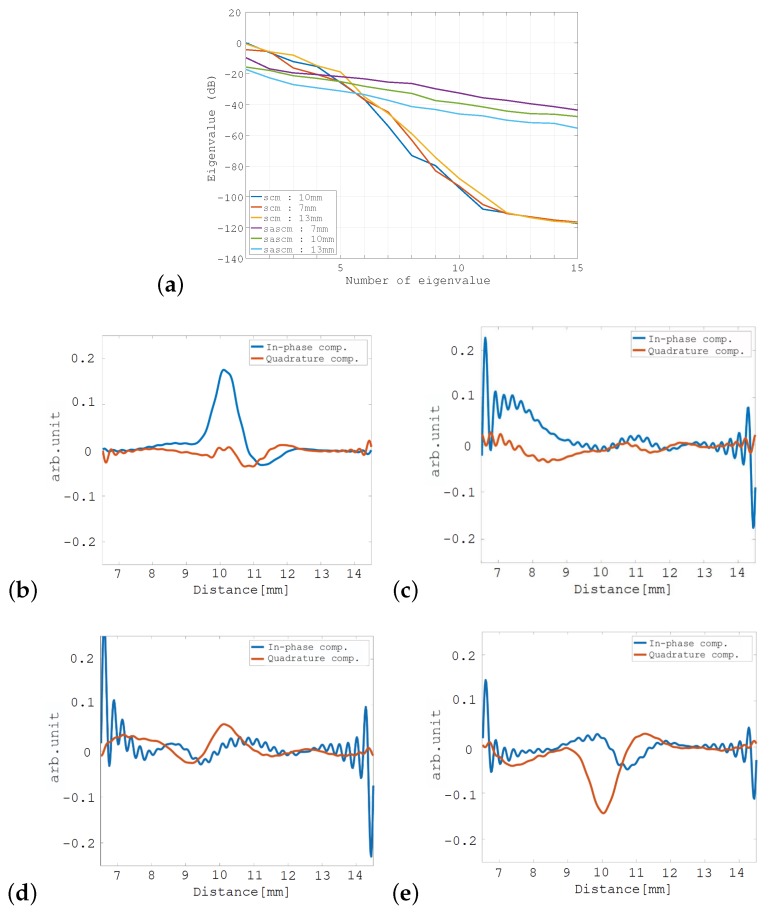
Eigenvalues and eigenvectors obtained for the point scatterer: (**a**) eigenvalue distributions of the SCM and the SA-SCM that were caused by changes in the distance from the point scatterer to the transducer; (**b**) the first eigenvector and (**c**) the second eigenvector of the SCM; (**d**) the first eigenvector and (**e**) the second eigenvector of the SA-SCM. In (**b**–**e**), a point scatterer is placed 10 mm away from the transducer, and the blue line indicates the in-phase (I) component, while the red line indicates the quadrature (Q) component.

Figure 6a shows the eigenvalue distributions for both the SCM and the SA-SCM for a point scatterer placed at distances of 7 mm, 10 mm, and 13 mm away from the transducer. These eigenvalues correspond to a beamformed echo passing through the point scatterer. The horizontal axis in this figure represents the eigenvalue number when the eigenvalues are arranged in descending order, while the vertical axis represents the eigenvalue magnitude. These results show that while the eigenvalue distributions of the two methods were not substantially dependent on the target position, they were found to differ greatly. In particular, the eigenvalues that correspond to the noise subspace were large for the SA-SCM, which is undesirable for application of the MUSIC algorithm.

**Figure 7:**
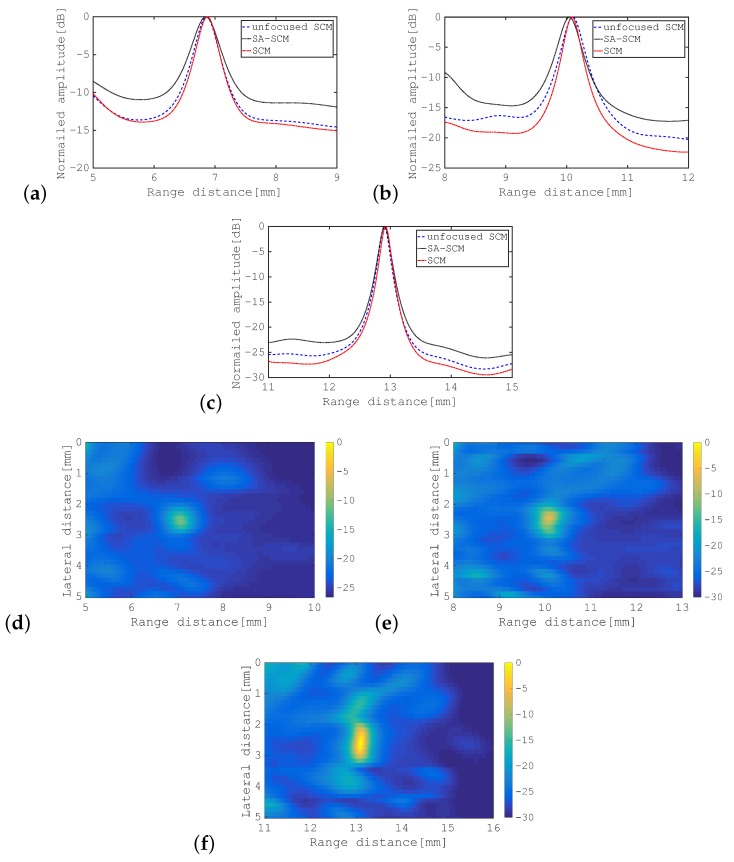
Cross-sectional profiles and B-mode images for the point scatterer when using *D* = 5. Cross-sectional profiles acquired using SCM, SA-SCM, and unfocused SCM with the transmission position fixed at the center. The point scatterer was placed at: (**a**) 7 mm; (**b**) 10 mm; and (**c**) 13 mm. B-mode images of the point scatterer acquired using the SA-SCM at positions of: (**d**) 7 mm; (**e**) 10 mm; and (**f**) 13 mm. The color bar shows the dynamic range in dB.

Figure 6b,c show the eigenvectors corresponding to the first eigenvalue and the second eigenvalue in the SCM, respectively, where the I component is in blue and the Q component is in red in each case. Figure 6d,e show the corresponding eigenvectors for the SA-SCM. Comparison of these eigenvectors confirmed that, in the SA-SCM, the signal component was largely contained in the second eigenvector and was also included within the first eigenvector. While, in the SCM, the signals can only be confirmed for the first eigenvector. For a point scatterer, the signal subspace should initially be one-dimensional, and the spread of this signal subspace for the SA-SCM has an adverse effect on the enhanced axial resolution characteristics. The cross-sectional profiles of the SCM and the SA-SCM for various distances to the point scatterer are shown in Figure 7a–c. The method that is referred to as the “unfocused SCM” that is also shown in this figure will be defined in the following subsection. B-mode images are also shown for the SA-SCM in Figure 7d–f. These results show that the undesirable eigenvalue distribution of the SA-SCM disturbed the enhanced axial resolution processing.

In the simulations performed for this study, *D* was fixed at five on the premise that the number of scatterers was not known beforehand. However, because it is appropriate theoretically to set *D* = 1 for a single point scatterer, the calculation was performed when *D* = 1as a performance evaluation under the optimum conditions. As a result, there were no significant differences in the enhanced axial resolution performances at all target positions, whether *D* = 1 or *D* = 5. However, the corresponding evaluation when *D* is increased further will be important and is regarded as a task for future work.

#### 4.1.3. Effect of Transmission Path

The main differences between the SCM and the SA-SCM were the transmission path and the transmission strength. In this subsection, we focus on the former and evaluate its effects on performance. In the SA-SCM, the pulse propagation in different directions reduced the phase coherence in the beamforming, which may then cause performance degradation. Therefore, we proposed a restricted SA-SCM (unfocused SCM) that transmits divergent pulses multiple times from the same transducer position and evaluated its characteristics.

**Figure 8:**
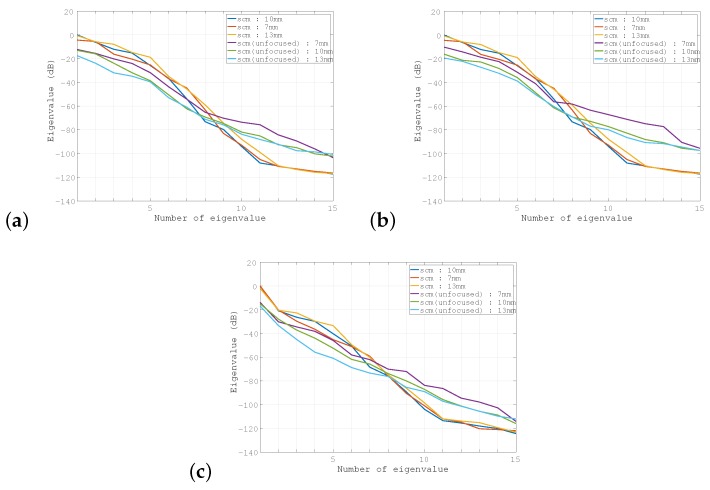
Eigenvalue distribution acquired using the unfocused SCM. For the point scatterer, the transmission position was fixed at: (**a**) the center of the array transducer and (**b**) the side edge of the array transducer; (**c**) shows the results for the line-type object with the transmission position at the center.

Figure 8a shows the eigenvalue distributions for the unfocused SCM with multiple transmissions from the center of the transducer to a point scatterer placed at three different positions, which are superposed on the corresponding distributions of the SCM. Similarly, Figure 8b shows the eigenvalue distributions for the unfocused SCM for transmissions from the side edge of the transducer. These results show that the eigenvalue distributions for unfocused SCM tended to be the same as those of SCM when all transmissions were launched from the same position. This trend has been shown to be stronger when the transmission is launched from the center of the transducer. Figure 8c is explained in the following subsection. The cross-sectional profiles produced using the unfocused SCM with transmissions from the center are superimposed on Figure 7a–c.

#### 4.1.4. Effect of Divergent Wave

The sound pressure of a divergent pulse is weaker than that of a focused pulse, which reduces the SNR of the received echo. This subsection evaluates the adverse effects of these low SNR characteristics on the performance degradation of the SA-SCM. To improve the SNR implicitly, a line-type target was used in this case (Figure 3a). The target had a width of 2 mm along the lateral direction and had one mesh for the FEM calculations along the range direction.

**Figure 9:**
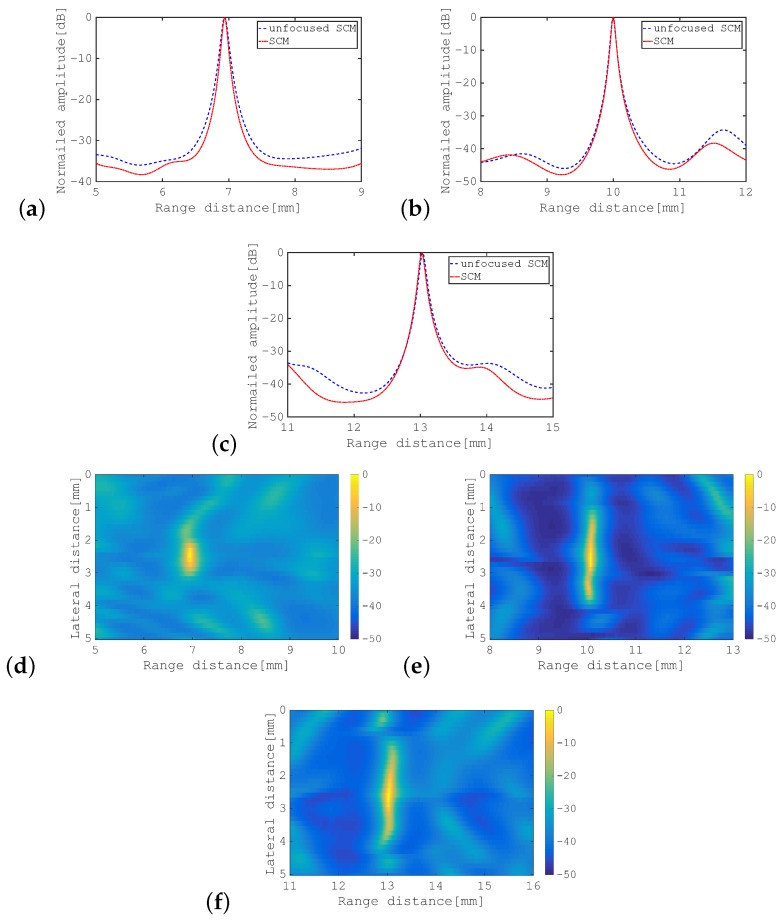
Cross-sectional profiles and B-mode images for line-type scatterer. Cross-sectional profiles acquired using the SCM and the unfocused SCM with the transmission position fixed at the center. The line-type scatterer is placed at distances of: (**a**) 7 mm; (**b**) 10 mm; and (**c**) 13 mm. B-mode images of the line-type scatterer acquired using the unfocused SCM for positions of: (**d**) 7 mm; (**e**) 10 mm; and (**f**) 13 mm. The color bar shows the dynamic range in dB.

In Figure 8c, the eigenvalue distributions of the unfocused SCM for transmission from the center of the transducer to the line-type scatterer at three positions, i.e., 7 mm, 10 mm, and 13 mm, are superposed on those of the SCM. Comparison of Figure 8c with Figure 8a,b shows that the eigenvalue distribution of the unfocused SCM for a line-type object was closer to the corresponding eigenvalue distribution of the SCM than those for a point-type object that were produced using the unfocused SCM, as shown in Figure 8a,b. This indicates that the differences in both the transmission path and the SNR between the SCM and the SA-SCM caused degradation of the enhanced axial resolution performance for the SA-SCM. Figure 9a–c shows the cross-sectional profiles acquired using the SCM and the unfocused SCM. The B-mode images acquired using the unfocused SCM are shown in Figure 9d–f. In particular, Figure 9a–c indicates that the weakening of the propagating pulse intensity in the SA-SCM had a strong effect on the range resolution.

### 4.2. Results of Experiments

**Figure 10:**
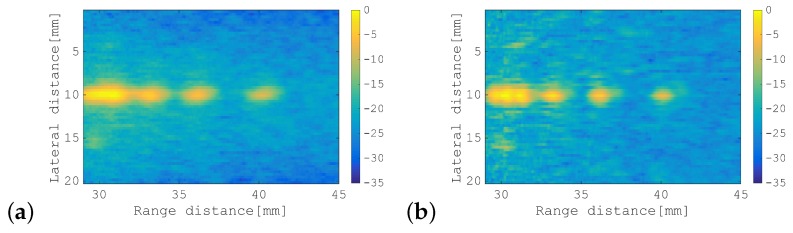
B-mode images of phantom acquired using: (**a**) SA-PCT; and (**b**) unfocused PCT. The color bar shows the dynamic range in dB.

**Figure 11:**
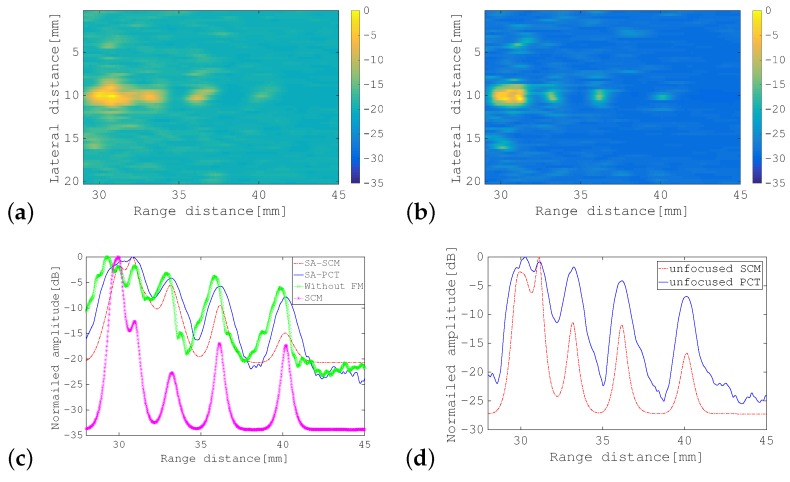
B-mode images of phantom acquired using: (**a**) SA-SCM; and (**b**) unfocused SCM. Amplitude profiles along the range direction of: (**c**) SA-PCT, as shown in Figure 10a, and the SA-SCM, as shown in (**a**); and (**d**) the unfocused PCT, as shown in Figure 10b, and the unfocused SCM in (**b**). For comparison, (**c**) also shows results acquired using a focused FM chirp pulse with the wide band pulse and the SCM. All eigenvalues that were greater than the minimum eigenvalue by more than 15 dB were selected to correspond to the signal subspace. The color bar shows the dynamic range in dB.

**Figure 12:**
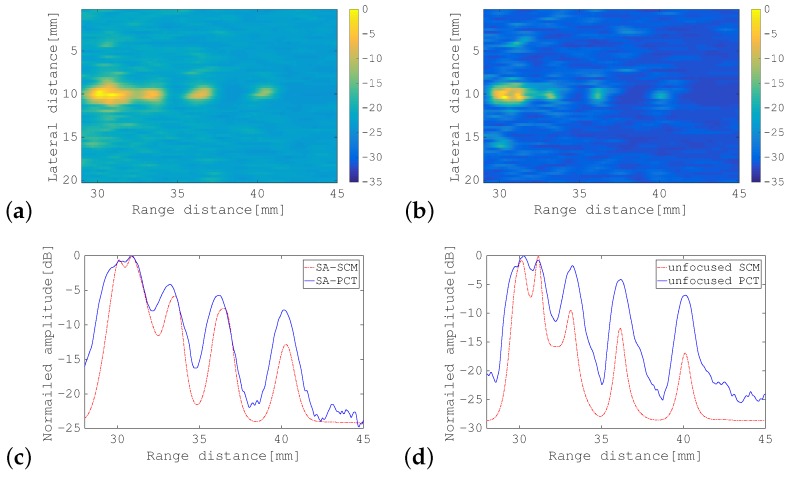
B-mode images of phantom acquired using: (**a**) SA-SCM; and (**b**) unfocused SCM. Amplitude profiles along the range direction for: (**c**) SA-PCT as shown in Figure 10a and the SA-SCM in (**a**); and (**d**) the unfocused PCT shown in Figure 10b and the unfocused SCM in (**b**). All eigenvalues that were greater than the minimum eigenvalue by more than 10 dB were selected to correspond to the signal subspace. The color bar shows the dynamic range in dB.

**Figure 13:**
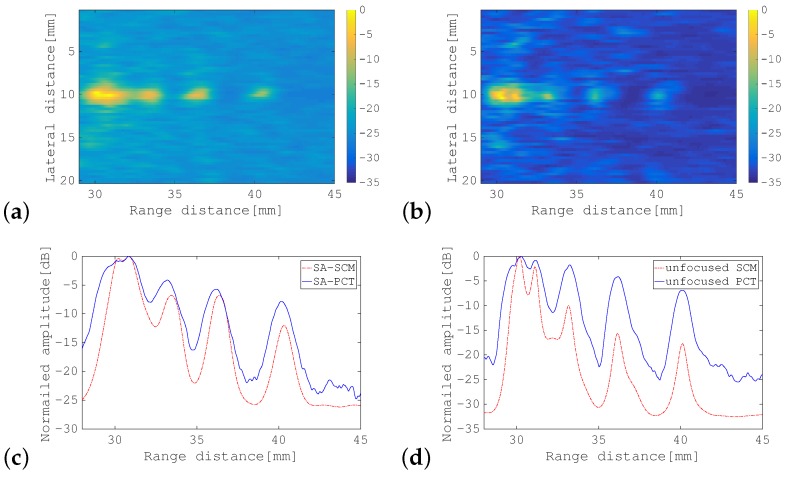
B-mode images of phantom acquired using: (**a**) SA-SCM; and (**b**) unfocused SCM. Amplitude profiles along the range direction for: (**c**) SA-PCT shown in Figure 10a and the SA-SCM in (**a**); and (**d**) the unfocused PCT shown in Figure 10b and the unfocused SCM in (**b**). All eigenvalues that were greater than the minimum eigenvalue by more than 5 dB were selected to correspond to the signal subspace. The color bar shows the dynamic range in dB.

**Figure 14:**
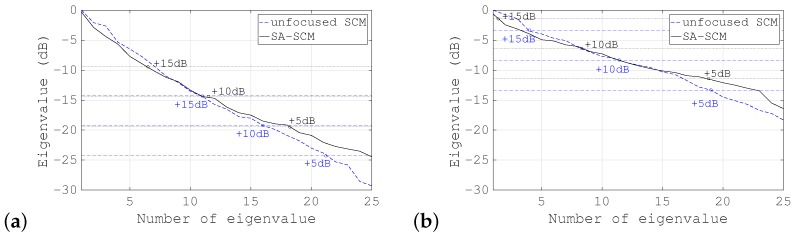
Eigenvalue distribution at (**a**) the center line, which contains targets, and (**b**) at another line with no targets.

First, the SA-PCT image and the unfocused PCT image are shown for comparison in Figure 10a,b, respectively. Figure 11, Figure 12 and Figure 13 show the results of processing using the SA-SCM and the unfocused SCM. As explained in Section 2.2, the dimension *D* of the signal subspace corresponds theoretically to the number of point scatterers. In this experiment, *D* = 6 was optimal, but because its effects are not generally understood, it was important to investigate the effects of the value of *D* on the image resolution. The images were therefore compared by varying *D* with reference to the eigenvalue magnitude of $\hat{R}$. Figure 11 defines the dimension *D* for the signal subspace, while assuming that all eigenvalues that are 15 dB larger than the minimum eigenvalue correspond to the signal subspace. Similarly, Figure 12 shows a corresponding increase in the eigenvalue of 10 dB, while Figure 13 shows a similar 5 dB increase to be used as a reference for signal subspace determination. In these figures, (a) shows the results for the SA-SCM and (b) shows the results for the unfocused SCM. In addition, (c) and (d) show the amplitude intensities at the center lines where the targets exist, with (c) corresponding to (a) and (d) corresponding to (b), respectively. The amplitude profiles from Figure 10a,b are superimposed on (c) and (d), respectively. In Figure 11c, the amplitude profile that corresponds to a pulse with a 4 MHz wide bandwidth without FM modulation and the profile obtained via the SCM using the focused FM chirp pulse are also superimposed. In the experiment, the intensity of the farthest target was greatly reduced because of the attenuation of the propagation medium, and the echo intensity for any later scatterer was lower than that of the scatterer that preceded it; therefore, the SCM processing result may not be identified so easily in this case, and the resolution may not be as good as had been expected. Figure 14a shows the eigenvalue distributions from the SA-SCM and the unfocused SCM that correspond to the line on which the targets exist, and Figure 14b shows the corresponding distributions for another line without targets. The thresholds that were used for the signal subspace determination are shown together in these figures.

These results show that it is difficult to separate the six targets completely, but it can be confirmed that both the SA-SCM and the unfocused SCM provided higher resolution in the range direction than the simple B mode images. The SA-SCM and the unfocused SCM also both offered high speckle suppression performance. Overall, it can be seen that a smaller *D* value, i.e., better resolution, corresponds to a higher threshold for the eigenvalue. We can also confirm that the unfocused SCM provides better resolution than the SA-SCM, which is consistent with the simulation results. If, unlike the cases of the simulations and experiments described in this paper, the scatterers are distributed throughout the imaging area, the performance of the unfocused SCM may be degraded.

It should be noted from Figure 11c that the SCM obviously had high resolution characteristics in the range direction, that is the effectiveness of the SCM processing to realize enhanced axial resolution can be seen in the figure.

## 5. Discussion and Conclusions

Firstly, from Figure 7, it should be noted that the range resolution was lower for both methods when the target and the transducer were closer together. This seems to be due to low coherence after beamforming over a short distance. This phenomenon is expected to be remarkable, particularly when imaging nearby targets with the SA-SCM, because the variation in the angle of incidence of the transmitted pulse becomes larger.

The most important result acquired from this study is the detailed analysis of the performance degradation of the SA-SCM when compared with that of the SCM. This degradation was found to be caused by mainly weakening of the transmitted wave intensity and deterioration of the phase coherence that is required for accurate reception beam forming, because the SA-SCM uses divergent pulses rather than the focused beams that are used in the SCM.

In the above evaluation process, we proposed the unfocused SCM with a fixed pulse transmission position to avoid coherence degradation during receive beamforming and compared its performance with the SA-SCM. The performance of the unfocused SCM was confirmed to be inferior to that of the SCM, but was shown to be far superior to that of the SA-SCM through both simulations and experiments. In the eigenvalue distribution (Figure 8c), there also was a slight difference between the SCM and the unfocused SCM, and it is considered that factors other than the above two issues exist.

Comparison of the cross-sectional profiles for SCM, SA-SCM, and unfocused SCM, as shown in Figure 7a–c, confirms that the unfocused SCM gave results that were much closer to those of SCM than SA-SCM. In addition, the results show that a shorter distance to the scatterer corresponded to lower resolution for the SA-SCM when compared with that of both the SCM and the unfocused SCM. As the scatterer came closer to the transducer, the phase coherence decreased because of the difference in the transmission position, so we can understand this result. The unfocused SCM worked well when the divergent pulses were transmitted from a centrally-located subarray in the transducer, but may be because the target was also centered. The unfocused SCM, unlike the SA-SCM, adopted transmission only from one direction, so the lateral resolution of the unfocused SCM for scatterers at various positions was expected to be lower than that of the SA-SCM. Montaldo et al. [19] proposed a plane wave compounding method to improve the SNR of the SA using divergent waves and pointed out that the multidirectional transmission was effective for image resolution through that method. In biological imaging, scatterers are distributed over the whole sample area. If this reduction is noticeable, it will be necessary to solve that problem.

In the phantom experiments, the resolution of the SA-SCM was worse than had been expected. When the scatterers were located close to the transducers, the time delay difference between the elements of the received echoes was large, so it is believed that the phase alignment at the time of receive beamforming was not appropriate. In contrast, in distant scatterers, propagating pulses are distorted by the effects of forward scattering by the scatterers that exist before them. In addition, we confirmed via phantom experiments that SCM clearly showed a superior performance when compared with SA-SCM. In contrast, unfocused SCM showed performance close to SCM, and it can be seen that the improvement of coherence was actually effective.

In this study, a simple DAS method was applied to beamforming, although improvement of the lateral resolution was not considered here. Recently, the various beam forming methods that are available to improve the lateral resolution have been researched vigorously [20,21,22,23,24]. When a high performance beamforming method is integrated with the SCM, simultaneous enhanced axial resolution properties can be expected in both the range and lateral directions.

The enhanced axial resolution method based on the SCM is a suitable method for imaging of scatterers with high reflectance in applications such as those targeting the boundaries of organs or blood vessel walls. Furthermore, small scatterers within these tissues appear as speckle patterns in B mode images and are useful not only for diagnosis of the tissue characteristics but also for image processing operations, such as motion analysis. We are currently investigating a method for use with small scatterers based on empirical Bayes deconvolution [12]. By combining this method with the SA-SCM, it will be possible to realize a new imaging scheme.

## Figures and Tables

**Table 1:** Parameter settings for the transmitted FM-chirp pulse.

Parameter	Value
Frequency band width	2 MHz
Chirp pulse duration	5 μm
Variation range of center freq.	4 to 6 MHz
Number of transmission	15
Apodization	Hanning window
